# Impact of mistreatment on the learning of novice medical students: An experimental study

**DOI:** 10.1111/medu.70103

**Published:** 2025-12-12

**Authors:** Fernanda Aparecida Tranches Martins, Ligia Maria Cayres Ribeiro, Rita de Cássia Corrêa Miguel, Telma Kremer, Alexandre Sampaio Moura, Silvia Mamede

**Affiliations:** ^1^ Professor Edson Antônio Velano University (UNIFENAS) Medical School Belo Horizonte Brazil; ^2^ University Medical Center Groningen, Wenckebach Institute (WIOO), Lifelong Learning, Education, Assessment and Research Network University of Groningen Groningen The Netherlands; ^3^ Independent Researcher Amsterdam The Netherlands; ^4^ Postgraduate Program in Medicine and Biomedicine, Faculdade Santa Casa Belo Horizonte Brazil; ^5^ Institute of Medical Education Research Rotterdam Erasmus Medical Center Rotterdam The Netherlands

## Abstract

**Introduction:**

Mistreatment toward peers, residents, and patients has been shown to trigger negative emotions among medical trainees. However, the impact of such experiences on trainees' learning needs to be further explored. This study reports on the impact of a situation of mistreatment experienced by a medical resident on novice medical students' learning of a scientific text.

**Methods:**

Videos portraying a medical resident receiving feedback about his performance in caring for a patient who died were used. Participants were randomly assigned to watch either a video where the feedback is accusatory and disrespectful (emotionally negative group‐ENG) or understanding and respectful (neutral group‐NG). Subsequently, all participants studied a scientific text. Study time and cognitive engagement with the text were recorded. Finally, they did a recall test about the text.

**Results:**

Data from 68 third‐year medical students were analysed. Test scores were lower for the students in the ENG compared with the NG [10.82 (5.37) and 14.44 (7.02), respectively, p = 0.020, *d* = 0.58]. No differences in cognitive engagement [3.98 (0.60) and 4.10 (0.73) for ENG and NG, respectively, p = 0.45] or time spent studying the scientific text [5:05 (1:36) and 4:56 (1:37) for ENG and NG, respectively, p = 0.71] were observed.

**Discussion:**

A simulated situation of mistreatment experienced by a resident negatively impacted the learning of a scientific text by novice medical students. These results extend the evidence on the negative impact of mistreatment on learning. It supports the relevance of mitigating mistreatment and adjusting training activities in situations of emotional distress.

## Abstract 2

**Mistreatment has ripple effects.** Even vicarious exposure can impair novice medical students' ability to learn, underscoring the hidden costs of toxic environments.

## 1 BACKGROUND

Experiencing negative emotions is part of the job of health professionals, as medicine is mostly about caring for individuals in moments of well‐being disruption.^1^ There is a source of negative emotions, however, that is not related to patient care and lies in the health professions training culture of mistreatment. Reports of public humiliation, physical and psychological abuse perpetrated by teachers, supervisors, peers, and patients have been extensively reported.^2^, ^3^, ^4^, ^5^, ^6^, ^7^, ^8^, ^9^ They have been shown to trigger negative emotions such as shame and sadness and to negatively affect students' well‐being.^6^, ^8^, ^10^, ^11^, ^12^, ^13^, ^14^ The impact of mistreatment on learning, on the other hand, has been poorly explored.^1^ This paper reports on the effect of a vicarious experience of mistreatment on novice medical students' learning of a scientific text.

Mistreatment encompasses a broad array of experiences such as discrimination, sexual harassment, belittlement, and verbal abuse.^7^ Surveys report a high prevalence of mistreatment in medical education,^2^, ^3^, ^4^, ^5^, ^6^, ^7^ and its consequences on well‐being and professional identity formation, such as depression and loss of confidence, have been extensively reported.^8^, ^9^, ^10^, ^11^, ^12^, ^13^, ^14^ Nevertheless, mistreatment can also be seen as a part of the socialisation of a doctor.^15^ Trainees might not define a disrespectful action as mistreatment if they think it is done with an educational purpose: some believe that ‘good intimidation’ would motivate improvement of skills, and also that what is learned under stress will not be forgotten.^15^, ^16^ Beyond ethical considerations of using mistreatment as a pedagogical tool, the effects of mistreatment on learning have been poorly explored. As mistreatment is a broad concept that encompasses different experiences, one approach to studying its effects on learning is exploring the emotions these experiences trigger.

Emotion can be defined as a transitory, usually intense, affective state triggered by specific events.^17^ It differs from affective traits, stable individual predispositions to emotional responses, and from affective mood, which lasts longer and has more unclear sources.^17^ Among the emotions reported by students who experienced mistreatment themselves or witnessed mistreatment towards others are anger, disgust, fear, and guilt.^13^ These are unpleasant feelings and therefore are classified as having negative valence, as opposed to the positive valence of feelings like joy and pride.

Studies from contexts other than medical education suggest that both negative and positive valence affective states can facilitate learning depending on the context.^17^, ^18^, ^19^, ^20^ Learning that requires active organisation and integrating new knowledge into one's previous knowledge structures, particularly relevant for medical training, benefits from a positive affective state.^17^, ^18^ On the other hand, a negative emotional state, such as anger triggered by social injustice, has been shown to facilitate learning of topics related to social inequalities.^19^

Studies in the medical education context also suggest that emotional experiences can influence learning. In a review article, McConnel and Eva^21^ found that the emotions experienced by medical students and residents during their training influence how these individuals identify, perceive, and interpret the information received. Positive emotions allowed learners to acquire more clinical skills and knowledge and fostered better doctor–patient relationships.

Negative emotions, on the other hand, have been shown to have negative effects on learning. In an experimental study by Kremer et al.,^22^ internal medicine residents watched a video in which a resident is mistreated, receiving inappropriate feedback after handling a critical situation. A control group watched a similar video where the resident received respectful feedback. Subsequently, all residents studied a scientific text, followed by a test about the text. The inappropriate feedback triggered negative emotions, and residents in this group had lower scores on the test. As residents in the mistreatment group spent less time studying the text, the negative effect on learning was attributed to an avoidance behaviour triggered by the mistreatment experience. While this study provides evidence of the negative effects of mistreatment when it is experienced by someone within one's own identity group, it remains unclear whether similar effects occur within different social groups.

Social identification shapes how we think, feel, and behave,^23^ and professional identity evolves throughout medical training. A novice medical student is not a ‘novice version’ of a medical resident, as both have different roles. The strains between training activities and learning, for example, are different. While students' roles are focused on learning, residents bear direct responsibility for patient outcomes.^24^, ^25^ As a result, these distinct groups are expected to perceive and navigate the clinical environment differently, which may influence how they interpret and react to situations of distress. Moreover, research in organisational identity has shown that social identification affects how individuals interpret and respond to acts of disrespect in the workplace.^26^ To what extent these factors affect learning in the clinical education context is yet to be studied.

We explored whether the learning of students at an early stage of training would be affected by a situation of mistreatment towards a medical resident. To examine this, we exposed novice students who had just initiated clinical care to a simulated situation of mistreatment experienced by a medical resident. A control group was presented with a neutral situation. Subsequently, the two groups were asked to study a scientific text. Study time, interest in the text, and free recall were compared between groups. We hypothesised that students exposed to the mistreatment situation would recall less information from the text compared with the neutral group. As the experience of mistreatment, different from Kremer et al.,^22^ targeted more novice trainees, we did not have a priori hypotheses regarding its effects on students' interest and engagement with the text.

## 2 METHODS

### 2.1 Participants

All 200 third‐year medical students from Professor Edson Antonio Velano University‐UNIFENAS, Brazil, were invited to participate. UNIFENAS has a six‐year curriculum with the two final years dedicated to clerkships. These students were chosen because at this stage of their training they begin their clinical experience in a hospital setting, where emotionally challenging situations are common. We assumed they would recognise the situation portrayed in the video as realistic. The sample size was calculated based on the large effect size observed in a similar study by Kremer et al.^22^ Forty‐six volunteers would be needed for a power of 0.9 and a significance level of 0.05. To account for dropout or missing data, our final target was 60 participants.

### 2.2 Design

The study had three tasks: priming, learning, and testing. In the priming task, participants were randomly assigned to watch a simulated interview conducted by members of an intra‐hospital committee providing feedback to a resident either in an accusatory and aggressive manner (emotionally negative group) or compassionately and politely (neutral group). Subsequently, all participants studied the same scientific text about a medical topic (learning task) and did a recall test about this text (test task). Students' scores on the recall test were taken as measures of learning about the text.

### 2.3 Procedure

The experiment was run online, using Flexiquiz software, which automatically randomised participants to the experimental groups, controlled the flow of the experiment, and recorded participants' answers. Sessions were run in computer labs and supervised by a researcher to support students and prevent communication between them during the experiment.

In the priming task, which was intended to trigger different emotional states across groups, students watched a 4‐minute video. Each group was shown a different version, produced for the studies by Kremer et al., and granted by the authors to be used in this experiment.^22^ The videos depicted simulated interviews conducted in a meeting room with three experienced physicians, members of an intra‐hospital committee, and a younger resident. The committee requested the resident to detail the care provided to a 40‐year‐old patient diagnosed with acute myocardial infarction who died in the emergency department. In the emotionally negative video, the committee members interviewed the resident in an accusatory and aggressive manner. They questioned him about a complaint filed by the patient's family, suggesting that the care he provided to the patient was delayed, that tests took too long to be reviewed, and that the diagnosis was delayed. The resident's attempts to provide his perspective were repeatedly interrupted. At the end of the video, the committee of physicians expressed that the family was right in considering that the patient did not receive adequate care and dismissed the resident in a harsh manner. The accusatory tone, unfair treatment, including the resident being denied the opportunity to defend himself, and the premature judgement of the care he provided to the patient, all delivered by a committee of senior doctors, are elements of mistreatment. In the neutral video, the interview was conducted in a compassionate and polite manner. The committee explained that they needed to understand what happened during the care, as the family was distressed about the patient's death. The resident was allowed to explain his perspective on the case and the context in which it happened. The interview concluded with the committee thanking the resident and stating that the care provided followed the service protocols. After watching the video, all participants answered three questions to identify whether the situation was realistic and effective in triggering different emotions on 5‐point Likert scales: (1) ‘Does the video portray a realistic situation?’ (*totally disagree* to *totally agree*), (2) ‘How do you feel at this moment?’ *(very bad* to *very well*) and (3) ‘How would you describe the emotions experienced by the resident during the feedback?’ (*very unpleasant* to *very pleasant*). Subsequently, all participants studied a medical text describing the diagnosis and causes of hypoglycaemia. This topic was chosen because students are familiar with it, without having yet mastered it. The instructions asked participants to study the text carefully. The software automatically recorded study time. Participants also completed the Situational Cognitive Engagement Scale to assess their engagement with the learning tasks, which has been previously shown to have good reliability as determined by Hancock's coefficient of 0.93 (cut‐off value of 0.70).^27^ The scale consists of items such as ‘I found this topic interesting’ and ‘I was totally focused on this task’. Responses to the items were based on a 5‐point Likert scale: 1—*not at all true for me*; 2—*not true for me*; 3—*neutral*; 4—*true for me*; 5—*very true for me*. The measurements of study time and situational cognitive engagement were used to analyse the level of involvement with the task at hand, with the first being a measure of behavioural and the second of cognitive engagement.

Finally, in the test task, participants were asked to write down everything they remembered from the text. Students could not move backward across tasks.

Sex, age, intended medical specialty, previous engagement in undergraduate courses, and information about personal history of anxiety and depression were collected to check if the randomisation strategy was successful.

The study was conducted as an extracurricular activity in two separate sessions to accommodate the volunteers' schedules. Approval was obtained from the UNIFENAS Ethics Committee (CAAE: 67038823.6.0000.5143), and students provided written consent. Participation was anonymous, and no identifiable data were collected.

### 2.4 Analysis of students' recall

The number of correct idea units (IU) presented in students' texts was taken as a measure of their learning of the scientific text. Initially, two templates with the IU presented in the text about hypoglycaemia were independently created by two researchers. The process consisted of breaking down the text into words or phrases containing relevant information. For example, ‘most cases of hypoglycaemia occur in patients with diabetes and are caused by insulin or other medicine, especially sulfonylureas’ was broken into (1) most cases of hypoglycaemia occur in patients with diabetes, (2) some medicine can cause hypoglycaemia, (3) insulin can cause hypoglycaemia, and (4) sulfonylureas can cause hypoglycaemia. The researchers then compared their templates and resolved discrepancies. Based on the final template, two certified internists independently scored 20% of the students' responses. Interrater reliability was measured using the intraclass correlation coefficient (ICC) for average measures, two‐way mixed model, and consistency, which was considered adequate (ICC = 0.91; 95% CI 0.73–0.97).^28^ The remaining texts were scored by the first author. The text scoring procedure was blinded to experimental condition.

### 2.5 Statistical analysis

Descriptive measures were presented to describe the results of the studied variables.

As *z* scores higher than 2.5 fall outside the range expected for 99% of a population, participants with *z* scores above this threshold on any of the study's primary outcomes—namely, text study time, situational cognitive engagement scale, and knowledge test score—were excluded from the analyses. Student's *t* tests (*t*) and Mann–Whitney tests (*U*) were used, as appropriate, to compare groups on continuous variables, and Pearson's chi‐square tests were used to compare categorical variables. Effect sizes were estimated by Cohen's *d*. The significance level was set at 95%.

## 3 RESULTS

Seventy‐two (36%) of the students invited volunteered to participate in the study. Four students were excluded from the analyses: two for not completing the tasks, one for having a *z* score higher than 2.5 in situational cognitive engagement (neutral group), and one for having a *z* score higher than 2.5 in study time (emotionally negative group). Sixty‐eight participants were included in the analyses (n = 68), divided into two groups of 34 students each: Emotionally Negative Group (ENG) and Neutral Group (NG).

### 3.1 Analysis of randomisation effectiveness

Table 1 presents the sociodemographic information of participants. Most of the volunteers were female students, reflecting the overall composition of the invited student pool, in which 64% were female. There were no differences between groups regarding sex, age, history of anxiety and depression/use of medicine to treat these conditions, or intended specialty. However, there were differences regarding engagement in undergraduate courses prior to entering medical school (*X* ^2^ = 8.67, p = 0.03). There were more participants with a history of undergraduate studies in health professions in the NG relative to the ENG (respectively, 14.7% vs. 3%).

**TABLE 1:** Sociodemographic characteristics of participants according to experimental conditions. Percentages and standard deviations into brackets.

	Emotionally negative group (34)	Neutral group (34)	Overall (68)
**Gender**			
Female	26 (76.5%)	23 (67.6%)	49 (72.1%)^a^
**Age**			
Mean (SD)	22.5 (2.6)	23.0 (4.0)	22.8 (3.4)^b^
**Intended specialty**			
Clinical	18 (54.5%)	17 (50%)	35 (52.2%)
Surgical	9 (27.3%)	13 (38.2%)	22 (32.8%)
Other	6 (18.2%)	4 (11.7%)	10 (14.9%)
No information	1	0	1
**History of depressive or anxiety disorder**			
No	8 (24.2%)	15 (44.1%)	23 (34.3%)
Yes	24 (72.7%)	17 (50.0%)	41 (61.2%)
Do not know/Do not want to answer	1 (3.0%)	2 (5.9%)	3 (4.5%)
No information	1	0	1
**Use of anxiolytics, antidepressants, or mood stabilizers**			
No	22 (66.7%)	24 (79.6%)	46 (68.7%)
Yes	10 (30.3%)	10 (29.4%)	20 (29.9%)
Do not know/Do not want to answer	1 (3.0%)	0 (0.0%)	1 (1.5%)
No information	1	0	1
**Previous undergraduate experience**			
No	28 (84.9%)	27 (79.4%)	55 (82.1%)
Yes, in health care	1 (3.0%)	5 (14.7%)	6 (8.9%)
Yes, other	4 (12.0%)	2 (5.9%)	6 (9.0%)
No information	1	0	1

^a^ Target group: 64%.

^b^ Target group: 23.35 (3.95).

### 3.2 Analysis of the effectiveness of experimental manipulation

Both groups considered the situations depicted in the videos to be equally realistic [4.35 (0.65) and 4.41 (0.61) for ENG and NG, respectively, *U* = 603.0, p = 0.732]. Concerning the emotion experienced by the resident portrayed in the video, from the participants' perspective, the ENG described it as being more negative relative to the NG [1.21 (0.42) and 1.62 (0.65), respectively, *U* = 749.5, *p* = 0.005]. Similarly, regarding students' own emotions, the ENG also showed to be more negative relative to the NG [2.06 (0.79) and 2.62 (0.74), respectively, *U* = 761.5, p = 0.007] (Table 2).

**TABLE 2:** Mean scores (range 1–5) on the realism of the video, perception of resident's feelings and personal feelings according to experimental conditions. Standard deviations into brackets.

	Emotionally negative group (34)	Neutral group (34)	Overall (68)
Realism of the video	4.35 (0.65)	4.41 (0.61)	4.38 (0.62)
Perception of the resident's feelings	1.21 (0.42)	1.62 (0.65)	1.42 (0.58)
Perception of students' own feelings	2.06 (0.79)	2.62 (0.74)	2.34 (0.81)

### 3.3 Analysis of primary outcomes

**FIGURE 1:**
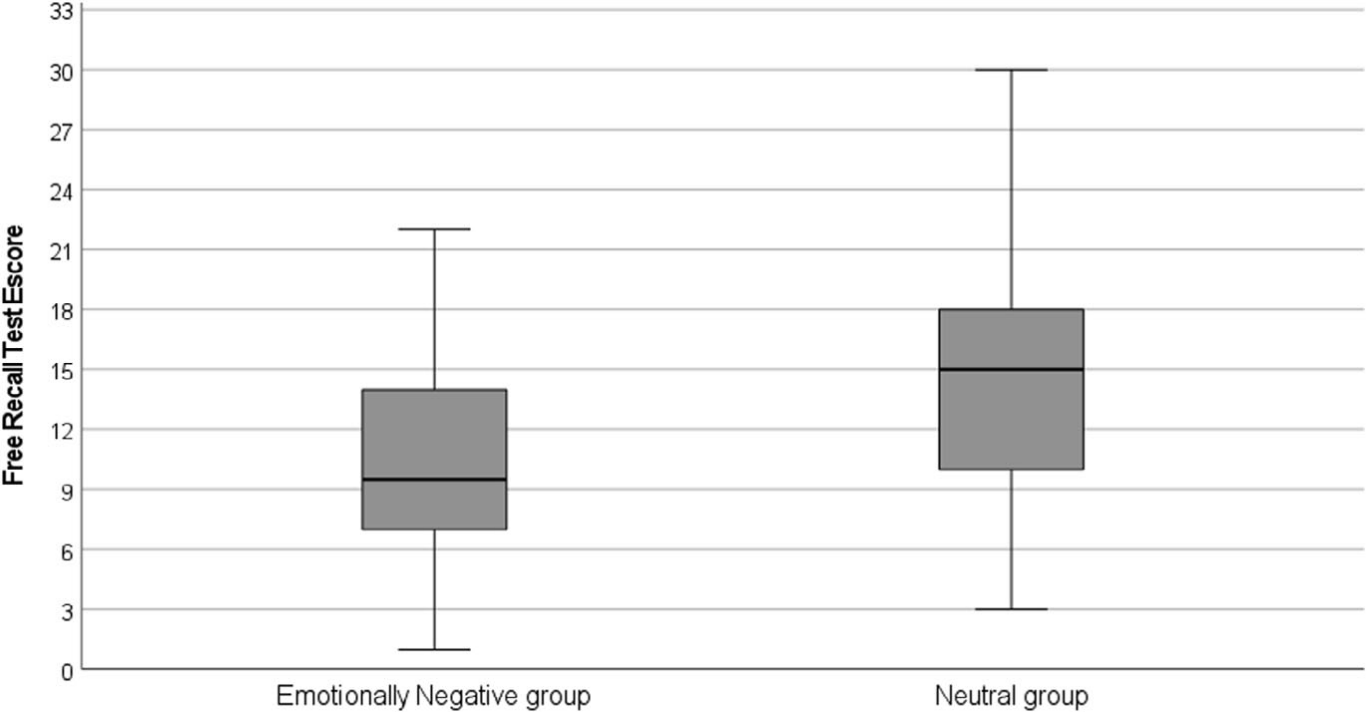
Free recall test scores according to experimental condition.

Table 3 shows the primary outcomes. There were no differences between groups regarding study time, [5:05 (1:36) and 4:56 (1:37) for ENG and NG, respectively, *t*(66) = 0.38, p = 0.71, mean difference = 0:08 (CI −0:38 to 0:55)] and situational cognitive engagement [3.98 (0.60) and 4.10 (0.73) for ENG and NG, respectively, *U* = 620.5, p = 0.45]. Finally, concerning the free recall test score, the ENG had lower scores compared with the NG [10.82 (5.37) and 14.44 (7.02), respectively, *t*(66) = 2.39, p = 0.02, mean difference = 3.61 (95% CI 0.59 to 6.64), *d* = 0.58 (95% CI 0.09 to 1.06)]. Figure 1 shows recall test outcomes.

**TABLE 3:** Mean study‐time, situational cognitive engagement, and test scores according to experimental conditions. Standard deviations into brackets.

	Emotionally negative group (34)	Neutral group (34)	Overall (68)
Study time (minutes)	5:05 (1:36)	4:56 (1:37)	5:00 (1:36)
Situational cognitive engagement (1–5)	3.98 (0.60)	4.10 (0.73)	4.04 (0.67)
Free recall test score	10.82 (5.37)	14.44 (7.02)	12.63 (6.46)

## 4 DISCUSSION

This study tested the effect of witnessing the mistreatment of a medical resident on novice medical students' learning. To do so, it compared the amount of information students recalled about a scientific text they studied just after watching a video of a resident receiving feedback about his clinical performance, either respectfully or disrespectfully. Watching the resident being mistreated negatively affected students' recall of the scientific text, with a medium effect size.^29^ This negative effect on learning was not mediated by students' engagement with the text.

The results align with our hypothesis and reproduce what has been previously observed outside and inside medical education. Negative emotions have been shown to hinder learning when organisation and integration of new knowledge into previous knowledge structures are needed.^17^, ^18^ This cognitive processing of information is needed for learning from a scientific text about a topic that students were familiar with but had not yet mastered, as was the case for the participants in this study. In the study of Ifenthaler,^18^ fake and negative feedback received by university students about their own performance hindered students' reasoning ability. Kremer et al.^22^ observed that watching a video of an internal medicine resident receiving disrespectful feedback hindered subsequent learning of scientific information by trainees at the same level and specialty of training. In our study, novice medical students just initiating their experience with patient care in a hospital were affected by a situation experienced by a resident, years ahead in medical training.

In the study by Kremer et al.,^22^ the residents primed to negative emotion spent less time studying the scientific text than those not primed. It is assumed that the shorter engagement with the text was possibly caused by an avoidance response, an unconscious reaction in which individuals seek to get away from a stimulus that triggers negative emotions. The shorter engagement with the text would have, then, hindered learning. In our study, however, both students in the ENG and NG dedicated similar study time to the text. The differences in the test scores observed, therefore, cannot be attributed to the same mechanism. The fact that both ENG and NG students reported similar levels of cognitive engagement with the scientific text, a measure of their interest in it, reinforces the possibility of a different mechanism operating.

One possible explanation for the lower test scores in the ENG group is a saturation of working‐memory capacity that these students might have experienced. Working memory is where new and prior knowledge are processed to create new knowledge structures. Its capacity is limited, and it is possible that the negative emotion triggered by the situation of mistreatment led to a rumination on the situation, similar to the thought patterns of escape observed when people experience fear and anxiety.^17^, ^20^ Fuks, for example, reports on a situation in which mistreatment toward a patient prevented a student from paying attention to an instructional activity about a biomedical topic.^9^ If students in our study, consciously or not, ruminated about the situation of mistreatment portrayed in the video, there would be less space in their working memories to process the biomedical information presented in the text, therefore hindering learning from it. An alternative explanation is that the situation of mistreatment triggered anxiety among students due to fear of experiencing similar situations. They aspire to become residents in the future and might also be experiencing power imbalances such as the situation illustrated in the video. This anxiety might have hindered retrieval of prior knowledge relevant to learning from the text, and/or the ability to focus attention on information that was new to them.^30^, ^31^ These explanations, however reasonable, require future investigation.

### 4.1 Educational implications

To the best of our knowledge, this is the first study to show that mistreatment can negatively affect novice medical students' learning. The negative effect was observed even when mistreatment was directed towards a much more senior colleague and portrayed in a video. It adds to the evidence on the negative effects of the culture of mistreatment in medical education, and the relevance to building efforts to change it. Simultaneously, while nurturing a culture of respect in medical education is of utmost relevance, it is not realistic to think that mistreatment and abuse will cease to exist. Acknowledging that disturbing episodes witnessed by the students can hinder the learning of information unrelated to the experience can help teachers manage such situations. Just asking students to suppress emotions and concerns is likely to be unsuccessful, as it might have the opposite effect of keeping the thought activated.^31^ Emotional regulation strategies, such as cognitive reappraisal, while showing positive results in contexts as when negative emotions are triggered by images, failed to counteract the negative effects of mistreatment within medical residents.^32^ On the other hand, evidence from stereotype threat research suggests that framing activities as challenges might be helpful.^33^ Providing guidance to students is a possible alternative approach, as feelings like shame and anger hinder self‐regulation abilities.^17^ Future research should explore such strategies in the context of mistreatment in medical education. Noteworthy, our study has provided additional support for a laboratory model that could be used for advancing in this research line.

### 4.2 Limitations

This study was run in a single medical school, and all participants were third‐year medical students. Generalisation to different audiences and contexts should, therefore, be careful. The study's findings inform only about the immediate effects of the vicarious experience of mistreatment on learning. How long such an effect would last is yet to be explored in future studies. For ethical reasons, we exposed students to vicarious, simulated mistreatment. While it can be seen as a limitation of the study, it is reasonable to assume that real‐life experiences would have a larger effect.

Our experimental groups were different in regarding students' experience in other health care undergraduate courses, with the NG having more students who had been exposed to health care training before medical school compared with the ENG. These more experienced participants could have more extensive previous knowledge about the text studied, which could have contributed to the better outcome of the NG on the recall test. The scores of the more experienced students in the NG, however, did not differ from those of the less experienced students in the same group. Even considering a *z*score cut‐off value of 2.0, a less strict value compared with the cut‐off of 2.5 used to define outliers in this study, there were no outliers within the NG, indicating that their performance on the test was similar to their peers.

Finally, although characteristics such as female students' proportion and mean age are similar between the target group and the volunteers, the potential for non‐response bias should still be considered.

## 5 CONCLUSIONS

This study observed that watching a video portraying a situation of mistreatment toward a resident affected novice medical students' learning of a scientific text. It adds to the evidence that challenges the perspective of blaming, shaming, and humiliating as valuable pedagogical tools. Future research should address how the emotional states triggered by such experiences affect students in different moments of training and evolve throughout time. Exploring how schools and teachers can support students coping with it, mitigating the negative effects that seem to carry over subsequent learning activities, is also much needed.

## AUTHOR CONTRIBUTIONS

**Fernanda Aparecida Tranches Martins:** Conceptualization; investigation; methodology; writing—review and editing; formal analysis; data curation; resources. **Ligia Maria Cayres Ribeiro:** Conceptualization; investigation; writing—original draft; methodology; writing—review and editing; formal analysis; data curation; supervision; project administration. **Rita de Cássia Corrêa Miguel:** Investigation; methodology; writing—review and editing; formal analysis; project administration; supervision; data curation. **Telma Kremer:** Conceptualization; writing—review and editing; methodology; resources. **Alexandre Sampaio Moura:** Conceptualization; methodology; writing—review and editing; project administration; supervision. **Silvia Mamede:** Conceptualization; writing—review and editing; methodology; supervision.

## ETHICS STATEMENT

This study was approved by the UNIFENAS Ethics Committee (CAAE: 67038823.6.0000.5143), and informed consent was obtained from all participants. A researcher was available to offer emotional support and address any feelings or concerns expressed by students after the data collection sessions.

## Data Availability

The data that support the findings of this study are available from the corresponding author upon reasonable request.
